# Progression of bilateral adrenal incidentalomas: a monocentric cohort study

**DOI:** 10.3389/fendo.2026.1912889

**Published:** 2026-09-03

**Authors:** Alessandra Mangone, Ilaria Pes, Sofia Frigerio, Riccardo Renda, Emanuele Ferrante, Erika Peverelli, Giovanna Mantovani, Serena Palmieri

**Affiliations:** 1 Department of Clinical Sciences and Community Health, University of Milan, Milan, Italy; 2 Endocrinology Unit, Fondazione IRCCS Ca’ Granda Ospedale Maggiore Policlinico, Milan, Italy

**Keywords:** adrenal incidentaloma, autonomous cortisol secretion, bilateral adrenal incidentalomas, cortisol excess, MACS

## Abstract

**Purpose:**

Bilateral adrenal incidentalomas (BAIs) are increasingly detected during abdominal imaging. Their initial clinical, hormonal, and radiological presentation is heterogeneous, and their natural history remains unclear. Current ESE/ENS@T guidelines lack specific recommendations for BAIs management.

**Methods:**

Retrospective single-center cohort study enrolling 185 consecutive non-operated patients referred between 2009 and 2023 to our endocrinology unit for BAIs (≥10 mm) with thorough assessment of hormonal, genetic and radiological data. Exclusion criteria were interfering comorbidities or incomplete data. Finally, 120 patients (63 females, mean age = 64.8 ± 8.2) were enrolled. Of these, 64 and 61 patients completed a minimum biochemical and radiological follow-up of 36 months, respectively.

**Results:**

At baseline, 56% of patients had mild autonomous cortisol secretion (MACS; 1-mg dexamethasone suppression test [1 mgDST] >1.8 µg/dl), and 44% had non-functioning (NF) BAIs. During follow-up, 32.1% of NF patients developed MACS (biochemical progression, BP), while 67.9% remained NF (biochemical stability, BS). Furthermore, 44.3% of patients showed radiological progression (RP) (increase in number and/or size of nodules ≥3 mm). The BP group had significantly higher baseline 1 mgDST values than the BS group (median: 1.3 vs. 1.1 µg/dl, *p* = 0.025). No other differences were detected between BP versus BS or RP versus radiological stability (RS) groups. In the RP group, only 7.4% of patients also showed BP. Patients with RP >10 mm (13.1%) had higher baseline 1 mgDST levels (3.5 vs. 2.2 µg/dl, *p* = 0.045).

**Conclusions:**

A non-negligible percentage of BAIs show hormonal and/or radiological progression. Our findings suggest that BAIs may represent a subgroup warranting individualized clinical reassessment and further prospective multicenter studies to define optimal surveillance strategies.

## Introduction

Bilateral adrenal incidentalomas (BAIs) are detected in approximately 15% of patients with incidentally discovered adrenal tumors (1–3). The initial presentation of BAIs is characterized by clinical, hormonal and radiological heterogeneity.

The most common adrenal incidentalomas are non-functioning adrenocortical tumors (NFATs), accounting for approximately 70%–85% of cases (4, 5). In NFATs, a conservative management approach is usually adopted, and one of the most debated topics in this context is the appropriate follow-up strategy for unresected tumors (6). A systematic review and meta-analysis including 2083 patients with NFATs found that, after a mean follow-up of over 3 years, only 4.3% demonstrated subsequent hormonal activity (7). However, the follow-up strategy for unresected NFATs remains controversial, especially for bilateral lesions, as the natural history of these tumors is still incompletely understood and data specifically focusing on BAIs are lacking. There are also no specific recommendations regarding the long-term management of BAIs, and the last ESE-ENS@T guidelines advise evaluating each adrenal lesion individually, applying the same diagnostic protocol as for unilateral masses (1). However, several studies have highlighted differences in hormonal and clinical characteristics compared to unilateral lesions (8, 9). In particular, up to 40% of BAIs are associated with mild autonomous cortisol excess (MACS), with a higher prevalence than in unilateral incidentalomas; yet diagnostic criteria for cortisol excess vary across studies (10). Identifying progression toward cortisol autonomy is essential, as MACS has been linked to an increased risk of mortality (11).

Primary bilateral macronodular adrenal hyperplasia (PBMAH) is a rare cause of Cushing’s syndrome and may be diagnosed initially as BAIs with MACS. There is currently no consensus on its definition: some authors suggest a combination of hormonal and radiological criteria (secretory profile and imaging features), while others consider histological confirmation essential (12). PBMAH is a complex and heterogeneous disease, and emerging evidence increasingly points to a genetic basis in a significant portion of reported cases (13). A progressive increase in hyperplasia and the development of macronodules has been described in PBMAH, sometimes accompanied by a gradual rise in cortisol secretion, eventually leading to Cushing’s syndrome and the need for therapeutic intervention (13).

To optimize the management of BAIs, it would be valuable to identify at the time of initial diagnosis which lesions are likely to evolve in terms of hormonal activity and/or radiological characteristics, and which are expected to remain stable, to define more personalized follow-up strategies. Our study aimed to investigate the natural history of a large monocentric cohort of patients with BAIs with a long follow-up of at least 36 months and to determine whether any clinical, biochemical, or radiological predictors of progression could be identified.

## Materials and methods

We retrospectively evaluated 185 consecutive non-operated patients who were referred between December 2009 and December 2023 to our Adrenal Diseases Tertiary Care Outpatients Clinic at Fondazione IRCCS Ca’ Granda Ospedale Maggiore Policlinico in Milan (Italy) for BAIs. Patients with the following were excluded: active malignancies/comorbidities (*n* = 7), drugs interfering with adrenal function (*n* = 5), bilateral myelolipomas (*n* = 2), primary aldosteronism (*n* = 5), congenital adrenal hyperplasia (*n* = 2), Cushing’s disease (*n* = 3), acromegaly (*n* = 2), hyperplasia without distinct nodules in one or both adrenals (*n* = 8), manifest bilateral macronodular adrenal hyperplasia at presentation, as defined by Bertherat et al. (13) (i.e., bilateral enlargement of the two adrenal glands and macronodules with internodular hyperplasia associated with cortisol excess; *n* = 4), morphologically different adrenal masses (*n* = 6), bilateral metastasis/hemorrhage/lymphoma (*n* = 3), bilateral pheochromocytomas (*n* = 1), and incomplete hormonal and/or radiological data (*n* = 17).

Finally, 120 patients (63 females, mean age 64.3 ± 8.4 years) with discrete BAIs (≥10 mm) were enrolled (**Table 1**). In patients with initial indeterminate radiological findings [i.e., tumor size ≥4 cm and/or unenhanced computed tomography (CT) Hounsfield Units (HU) >10], immediate additional imaging (contrast-enhanced CT or magnetic resonance) or a follow-up imaging in 6–12 months was performed to confirm the benign nature of the nodules. Hormonal, clinical, and radiological data were assessed at diagnosis and at the last visit. Patients with hormonal and/or radiological data available at least 36 months after diagnosis were considered for follow-up analyses.

**Table 1:** Baseline demographic, radiological and hormonal data of the study cohort of patients with bilateral adrenal incidentalomas (BAIs).

Demographic data	Entire cohort (*n* = 120)	Biochemical cohort (*n* = 64)	Radiological cohort (*n* = 61)	*p*-value
Sex, F/M (% F)	63/57 (52.5%)	37/27 (57.8%)	35/26 (57.4%)	ns
Median age (IQR), years	64 (59–70)	65 (61–71)	65 (60–71)	ns
Median BMI (IQR), kg/m^2^	26.8 (23.9–30.4)	26.8 (23.9–31.8)	26.7 (23.9–30.8)	ns
Smoking status, never/current/former, *N* (%)	23/36/29 (26/41/33%)†	13/15/21 (27/31/43%)†	14/13/19 (30/28/41%)†	ns
Radiological data				
Left: median *N* of nodules (range)	1 (1–3)	1 (1–3)	1 (1–3)	ns
Left: median diameter* (IQR), mm	18 (14–27)	17 (13.5–22)	18 (15–20)	ns
Right: median *n* of nodules (range)	1 (1–2)	1 (1–1)	1 (1–2)	ns
Right: median*(IQR), mm	18 (12–25)	16 (12–25)	18 (12–25)	ns
Hormonal data				
Median ACTH (IQR), ng/L	10.9 (7.9–17.1)	10.5 (7.6–16.6)	10.5 (8–16.6)	ns
Median cortisol (IQR), µg/dl	13.1 (11–16.1)	12.7 (10.9–16.1)	12.7 (11–15.5)	ns
Median 1mgDST (IQR), µg/dl	2.0 (1.3–3.3)	2.0 (1.25–2.8)	2.0 (1.3–2.9)	ns

N, number; F, female; M, male; IQR, interquartile ranges; BMI, body mass index; 1mgDST, cortisol after 1 mg dexamethasone suppression test; ns, not significant.

†Percentages calculated among patients with available smoking data (missing in 32/120 patients, 27%).

*For patients with multiple nodules, the diameter of the largest nodule was considered.

Adrenal hormone excess was defined according to the most recent ESE-ENS@T guidelines on the management of patients with newly diagnosed adrenal masses (1). Cushing syndrome was defined by the presence of typical clinical features together with suppressed ACTH levels and more than one positive screening test for hypercortisolism (late-night salivary cortisol, cortisol after overnight 1 mg dexamethasone suppression test (1 mgDST), and urinary free cortisol). MACS was defined by cortisol after 1 mgDST > 1.8 µg/dl on two different occasions in the absence of overt clinical features of Cushing syndrome. BAIs with normal 1 mgDST results were classified as non-functioning (NF). Biochemical progression during follow-up was defined as a change in the hormonal functional category, namely the transition from NF to MACS or overt Cushing syndrome. Because dehydroepiandrosterone sulphate (DHEAS) values were measured using different assay methods over the study period, values were categorized and analyzed according to sex- and age-specific reference ranges as either below the lower limit of normal (“low DHEAS”) or below the 25th percentile of the normal reference range.

Significant tumor growth was defined as an increase greater than 3 mm from the baseline measurement (14). As a sensitivity analysis, tumor growth was also assessed using the RECIST 1.1 criteria (>20% increase in the largest tumor diameter together with an absolute increase ≥5 mm) (1). Additionally, we reported data for patients whose tumors exhibited an increase in size exceeding 10 mm. Original radiological images were centrally reviewed by one of two endocrinologists with expertise in adrenal disorders. Whenever available, follow-up measurements were compared using the same imaging modality. Reviewers were not blinded to hormonal data.

As part of our clinical practice, even in the absence of overt PBMAH at baseline, patients with BAIs and MACS were offered genetic testing for *ARMC5* variants and a mixed-meal test to screen for ectopic gastric inhibitory polypeptide receptors (15), with a post-prandial increase in serum cortisol >50% from baseline used to define a positive response (16).

The local ethics committee approved this protocol study (Milan, 325_2021bis) and all patients provided written informed consent.

### Statistical analysis

Statistical analyses were conducted using STATA v.17 (StataCorp, College Station, Texas, USA) and GraphPad Prism version 9 (GraphPad Software, San Diego, CA, USA). Categorical variables are presented as numbers or percentages, while continuous variables are expressed as mean ± standard deviation (SD) if normally distributed, or median and interquartile ranges (IQR). Normality was assessed using the Shapiro–Wilk test. Continuous variables between groups were evaluated using the independent t-test for normally distributed variables and the Mann-Whitney U test for non-normally distributed variables. Categorical variables were compared using the chi-square test and Fisher’s exact test, as appropriate. Correlations between continuous variables were assessed using Spearman’s rank correlation coefficient. Comparisons between baseline and follow-up measurements within the same patients were performed using paired t-tests or Wilcoxon signed-rank tests, as appropriate. *P*-values < 0.05 were considered statistically significant.

## Results

The baseline demographic, radiological and hormonal data of the study cohort at enrollment are detailed in **Table 1**.

At baseline, 67 patients (56%) had MACS [cortisol after 1 mgDST >1.8 µg/dl], and 53 (44%) had NF BAIs. None had overt, clinically apparent Cushing syndrome. Overall, 8 out of 51 (15.7%) patients with MACS who were genetically screened had a pathogenic germline *ARMC5* variant. All MACS patients tested with a standard mixed meal (15) resulted negative.

Ninety-nine of 120 patients (82.5%) showed a single adrenal nodule in each gland at baseline, whereas the remaining patients (17.5%) had multiple nodules in at least one adrenal gland, without internodular adrenal hyperplasia.

Smoking status at baseline was available in 88/120 patients (73%): 23 (26%) never-smokers, 36 (41%) current smokers and 29 (33%) former smokers. Ever-smokers (current or former, *n* = 65) had a significantly larger baseline maximum tumor diameter than never-smokers (median 25 vs. 19 mm, *p* = 0.015), whereas no significant differences were observed between the two groups in terms of MACS prevalence, cortisol after 1 mgDST, ACTH, DHEAS, BMI, age, or number of nodules, nor in the rates of biochemical or radiological progression during follow-up (17).

### Biochemical progression

Sixty-four (53.3%) patients completed a minimum biochemical follow-up of 36 months, with a median follow-up time of 85 months (range: 36–264 months). Of note, the biochemical and radiological follow-up cohorts partially overlapped, with most patients completing both assessments and only 12 patients differing between the two cohorts. The baseline demographic, radiological, and hormonal data of this subgroup of patients are detailed in **Table 1**. No differences between groups (entire cohort, biochemical follow-up cohort, and radiological follow-up cohort) were found. No significant differences were also observed between patients who did and did not complete adequate follow-up concerning the main demographic, hormonal and radiological baseline characteristics (age, sex, BMI, MACS prevalence, 1 mgDST cortisol and ACTH levels, and tumor size).

At follow-up, nine of the 28 patients (32.1%) initially defined as NF-BAIs developed MACS (BP group), while 19 (67.9%) remained NF [biochemical stability (BS) group].

No patient with MACS at baseline progressed to clinically overt Cushing’s syndrome during follow-up. In this group, a slight, non-significant increase in 1 mgDST cortisol levels was observed, with a median of 2.6 µg/dl (IQR 2.1–3.8) at baseline and 2.9 µg/dl (IQR 2.3–3.9) at follow-up (*p* = 0.49).

**Figure 1:**
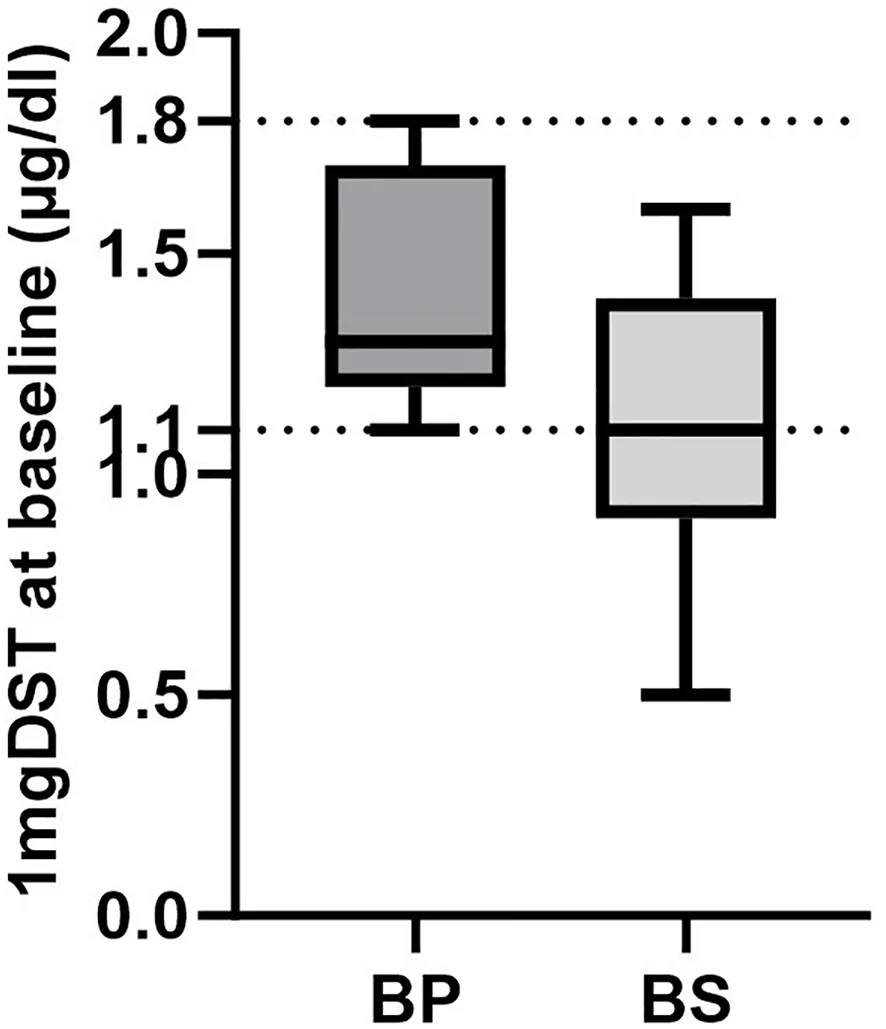
Differences in 1 mgDST levels at baseline among patients who showed biochemical progression (BP) or stability (BS).

Patients in the BP group showed significantly higher 1 mgDST levels at baseline (median: 1.3 vs. 1.1 µg/dl, *p* = 0.025) than patients in the BS group, as detailed in **Table 2** and shown in **Figure 1**. Interestingly, none of the BP group patients had a 1 mgDST < 1.1 µg/dl at baseline (1 mgDST range: 1.1–1.8 µg/dl in BP group vs. 0.5–1.6 µg/dl in BS group, see **Figure 1**).

**Table 2:** Differences in terms of baseline clinical characteristics between patients with non-functioning bilateral adrenal incidentalomas (NF-BAIs) who showed biochemical progression (BP group) and stability (BS group).

Clinical characteristics	BP group *N* = 9	BS group *N* = 19	*p*-value
Age, years	65 (62–71)	64 (58–72)	0.660
BMI, kg/m^2^	24.8 (23.3–30)	27.5 (24.2–32)	0.430
Ever-smoker, *N* (%)	6/8 (75)	10/13 (76.9)	1.000
Maximum diameter, mm	22 (18–29)	17 (15–27)	0.330
ACTH, ng/L	11 (9.9–12)	16.1 (8.9–22.5)	0.100
1mgDST, µg/dl	1.3 (1.2–1.6)	1.1 (0.9–1.4)	0.025
17OHP, µg/L	0.7 (0.4–0.7)	0.9 (0.3–1.1)	0.350
Low DHEAS, *N* (%)	0 (0)	1 (5.2)	1.000
DHEAS < 25th percentile, *N* (%)	2 (22)	5 (26.3)	1.000

Data are expressed as median and interquartile ranges (IQR) or numbers (N) and percentages (%).

1mgDST, cortisol after 1 mg dexamethasone suppression test; 17OHP, 17-hydroxyprogesterone; DHEAS, dehydroepiandrosterone sulphate.

Ever-smokers are defined as current or former smokers, and relative data are calculated only among patients with available smoking data.

No significant differences in terms of age, BMI, ACTH and 17-hydroxyprogesterone (17OHP) nor radiological presentation (baseline HU and maximum diameter) were detected at baseline between BP and BS groups, as detailed in **Table 2**. Moreover, no significant differences in DHEAS values were detected at baseline between the two groups (**Table 2**).

### Radiological progression

Sixty-one (50.8%) patients completed a minimum radiological follow-up of 36 months, with a median follow-up time of 81 months (range: 36–234 months). The baseline demographic, radiological, and hormonal data of this subgroup of patients are detailed in **Table 1**.

**Figure 2:**
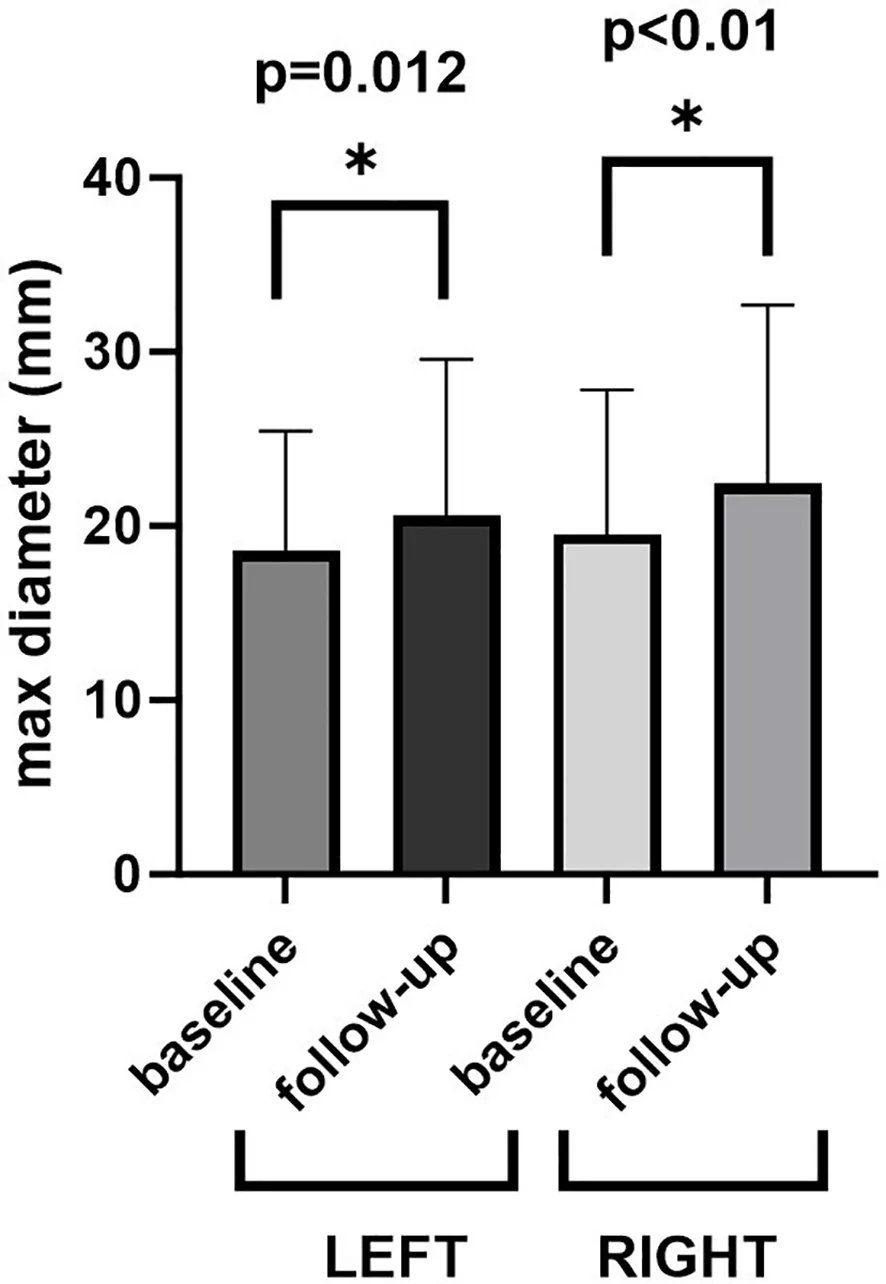
Comparison of maximum adrenal nodule diameter (mm) at baseline and follow-up. Mean maximum nodule diameter increased significantly over time in both the left (*p* = 0.012) and right (*p* < 0.01) adrenal glands. * indicates statistical significance (*p* < 0.05).

Overall, 27 out of 61 patients (44.3%) exhibited radiological progression (RP) during follow-up, defined as an increase in the number and/or size (≥3 mm) of adrenal nodules (14). Specifically, 25 patients (41%) showed an increase in the maximum diameter of nodules on both the left and right adrenal glands, while nine patients (14.8%) demonstrated an increase in the number of nodules. The overall increase in maximum nodule diameter between baseline and follow-up is illustrated in **Figure 2**. No significant differences in demographic, radiological, and hormonal parameters at baseline were detected between the RP and radiological stability (RS) group (**Table 3**).

**Table 3:** Differences in terms of baseline clinical characteristics between patients with bilateral adrenal incidentalomas (BAIs) who showed radiological progression (RP group) and stability (RS group).

Clinical characteristics	RP group *N* = 27	RS group *N* = 34	*p*-value
Age, years	66 (58–72)	64.5 (62–70)	0.27
BMI, kg/m^2^	26.7 (23.8–32.4)	27.1 (24.2–28.8)	0.47
Ever-smoker, *N* (%)	12/19 (63.2)	20/27 (74.1)	0.52
Maximum diameter, mm	18 (16–22)	16 (12–20)	0.34
ACTH, ng/L	10.6 (7.6–17.6)	10.5 (8.1–16.6)	0.61
1mgDST, µg/dl	2.4 (1.3–2.9)	1.8 (1.3–2.2)	0.37
17OHP, µg/L	0.7 (0.3–1)	0.7 (0.4–1)	0.15
Low DHEAS, *N* (%)	2 (7.4)	5 (14.7)	0.55
DHEAS < 25th percentile, *N* (%)	12 (44)	7 (20.5)	0.63

Data are expressed as mean and interquartile ranges (IQR) or numbers (N) and percentages (%).

1mgDST, cortisol after 1 mg dexamethasone suppression test; 17OHP, 17-hydroxyprogesterone; DHEAS, dehydroepiandrosterone sulphate; RP, radiological progression; RS, radiological stability.

Ever-smokers are defined as current or former smokers, and relative data are calculated only among patients with available smoking data.

In the RP group, only two patients (7.4%) had an associated BP from NF to MACS, five patients (18.5%) were already MACS at baseline, whereas the majority of RP group patients were NF at baseline and remained NF in the follow-up. As expected, all patients with a pathogenic germline *ARMC5* variant (*n* = 3) who completed the follow-up period showed an RP.

The longitudinal change in 1 mgDST cortisol (Δ1mgDST, follow-up minus baseline) did not differ between patients with RP and RS (median +0.25 vs. +0.33 µg/dl, *p* = 0.74). Likewise, the magnitude of nodule growth was not correlated with Δ1mgDST (*r* = 0.27, *p* = 0.22).

As a sensitivity analysis, applying the RECIST 1.1 criteria (1), 18/61 patients (29.5%) fulfilled the criteria for RP. The qualitative findings remained unchanged, with no significant baseline predictors of RP and only partial overlap with BP.

We then performed an additional sub-analysis of patients who exhibited an RP > 10 mm (8/61, 13.1%). Interestingly, among all the variables analyzed, we found higher baseline 1 mgDST levels in this subgroup compared with those with either RS or RP < 10 mm (median 1 mgDST 3.5 vs. 2.2 µg/dl, *p* = 0.045).

## Discussion

Bilateral adrenal masses are increasingly detected in routine medical practice. They are characterized by clinical, hormonal, and radiological heterogeneity, with some of them possibly representing the first manifestation of PBMAH.

More than half of patients with BAIs in the present study were found to have MACS at baseline, a higher prevalence than those registered in unilateral incidentalomas, as already underlined by previous literature (10). However, none had overt, clinically apparent Cushing syndrome. Genetic screening was recommended for all BAIs patients with MACS, and 15.7% were found to carry a pathogenic germline *ARMC5* variant. This finding has important clinical implications, as the early identification of PBMAH with pathogenic variants can guide a more personalized follow-up strategy, including screening for meningioma risk and early detection of affected family members of index cases (13). Moreover, all patients with MACS were tested with a mixed meal for ectopic GIP receptor screening, though no responses were registered.

The rest of our cohort (44%) was found to have NF BAIs. Current ESE-ENS@T guidelines suggest against further imaging studies and follow-up in patients with NFATs that show benign radiological features (1). While this strategy is cost-effective, reduces waiting times, and limits radiation exposure, it may lead to a loss of potentially relevant information, particularly in bilateral cases. These cases, although highly heterogeneous, appear to exhibit certain distinct characteristics (8, 9), including a notably higher risk of autonomous cortisol secretion, which has been associated with increased mortality (11). Furthermore, even in patients with NFATs, higher cortisol post-DST levels have been linked to greater cardiometabolic risk (18), and hypercortisolism is nowadays interpreted as a continuum, with higher cardiometabolic risk expected with higher cortisol post-DST.

The incidence of hormonal progression with the development of MACS from initially NFATs is relatively low in most studies. In a recent large longitudinal series by Ceccato et al. (19), none among 181 adrenal incidentalomas evaluated at follow-up progressed to Cushing’s syndrome, whereas 6.2% developed MACS after approximately 4 years. However, the reported rates are highly variable, reaching up to 31% in one study (20). This heterogeneity may be attributed to the lack of consensus on the diagnostic definition of MACS, especially in older studies (6). Interestingly, to date, no study has specifically analyzed the subgroup of patients with BAIs separately; rather, unilateral and bilateral cases have consistently been pooled together, potentially overlooking important clinical distinctions between these two entities. Notably, Falcetta et al. (21) and Araujo‐Castro et al. (22) identified the presence of bilateral lesions as a factor associated with the subsequent development of MACS. In our large cohort of BAIs, the incidence of BP to MACS was significant (32%), whereas no patient presenting with MACS progressed to clinically overt Cushing’s syndrome.

Previous literature also reveals considerable heterogeneity and lack of consensus regarding other predictive criteria for hormonal progression. An initially larger tumor diameter has been proposed as a potential predictor of MACS onset over time, with various threshold values explored across studies (19, 21, 23). However, this association has not been consistently confirmed by other authors (24–30). In our study, no differences in terms of radiological presentation were detected at baseline between the groups with and without BP.

Higher baseline cortisol levels after 1 mgDST have also been highlighted as possible indicators of risk of hormonal progression (20, 22, 25, 29, 31). In the above-mentioned study by Araujo‐Castro et al. (22), baseline post-1 mgDST cortisol levels were the most important factor for the development of BP, with rates of MACS development being 19.2, 32.3, and 68.1 cases/10,000 person-years for patients with 1mgDST at diagnosis < 0.9 μg/dl, 0.9–1.3 μg/dl, and > 1.3 μg/dl, respectively. Notably, in the study of Podbregar et al. conducted on a cohort of 67 NFATs, a 1mgDST cortisol threshold of 30 nmol/L (1.08 µg/dl) was identified as predictive of future MACS development (31). In our cohort, patients in the BP group similarly exhibited significantly higher 1 mgDST cortisol levels at baseline, and none of the patients in this group had a baseline 1mgDST value below 1.1 µg/dl.

Subnormal DHEAS levels were found only in 35% of cases of this series and did not reflect evolution toward autonomy. This is in line with a recent review by Bertherat et al. that emphasizes that DHEAS levels are often low but not completely suppressed in patients with PBMAH, in contrast with patients with unilateral cortisol-secreting adenoma (32). ACTH values were also not predictive of either biochemical or radiological progression in our work, in opposition to the study of Falcetta et al. previously mentioned, where the risk of hormonal progression was associated with the presence of low/suppressed ACTH values at the time of detection (21). A possible, though speculative, explanation previously suggested relies on the presence of local production of ACTH by the steroidogenic cells themselves in PBMAH. Indeed, aberrant hormone receptors have been proposed to regulate the paracrine secretion of ACTH in the literature, creating an autocrine/paracrine loop that may promote adrenal growth and steroid secretion in PBMAH (33).

Focusing on radiological progression, the study by Ceccato et al. (19) reported only minimal increases in tumor diameter after a median follow-up of 52 months, with significant growth observed in just 3.3% of cases. Considering other studies, the incidence of diameter increase is highly variable, ranging from 5% to 56.7%, and generally increases with the length of follow-up (6, 21, 27, 30). Notably, the criteria for nodular enlargement are often not reported, with changes as small as 1–2 mm potentially attributable to measurement errors (14). For this reason, we defined radiological progression using the threshold growth rate of ≥3 mm advised by Corwin et al. (14) to identify those patients requiring further workup or continued monitoring. We acknowledge that this relatively sensitive definition may also capture some degree of measurement variability, particularly given the retrospective design and the non-standardized imaging protocols over the study period. In our study, radiological progression in patients with BAIs was around 44%, a relatively high percentage that may partly reflect the possibility that some BAIs represent early manifestations of PBMAH, although this remains speculative in the absence of genetic or longitudinal phenotypic confirmation in most cases. Indeed, all three patients found to carry a pathogenic germline ARMC5 variant who completed follow-up showed radiological progression. Among other predictive criteria of radiological enlargement, previous literature (19) identified an initial diameter >2.4 cm to be associated with greater tumor growth over time, yet with conflicting results (29). In our cohort, no significant differences in demographic, radiological, or hormonal characteristics at baseline were found between the RP and RS groups. However, when considering a larger increase in nodular diameter >10 mm, higher 1 mgDST levels at baseline also predicted progression.

A potential link between hormonal activity and tumor growth has been previously suggested, but findings remain inconsistent (19–21, 23, 27). For example, Papanastasiou et al. (20) reported a correlation between adenoma size increase and the development of MACS, supporting the value of biochemical re-evaluation in cases of mass enlargement. However, this association has not been confirmed by other studies (28). In our analysis of BAIs, only 7.4% of patients in the RP group who were initially NF subsequently progressed to MACS.

Finally, since completion of the present study, two recent important contributions have further advanced the understanding of benign adrenal lesions. A large multicenter ENS@T study demonstrated that longitudinal changes in cortisol secretion are more common than previously recognized and showed that bilateral lesions were more prevalent among patients with persistent MACS, further supporting the hypothesis that BAIs may represent a distinct clinical subgroup (34). Moreover, a recent large Italian single-center study reported that patients with bilateral NFATs have a less favorable cardiometabolic profile than those with unilateral lesions (35), reinforcing the concept of bilaterality as a distinct clinical phenotype warranting further investigation.

This study has several limitations. First, the retrospective nature of data collection inherently carries potential biases and limitations, including non-standardized imaging protocols throughout the study period, which may have influenced the assessment of small changes in tumor size. Regarding the risk of BP, some degree of biological and analytical variability around the diagnostic threshold cannot be excluded. Moreover, given the limited number of progression events, multivariable analyses could not be reliably performed, and therefore the associations identified should be regarded as exploratory and not as independent predictors. Data on possible cortisol-related comorbidities were incomplete and collected at varying time points during follow-up; therefore, they were not included in the final analysis. Smoking status, a relevant cardiovascular risk factor that has been associated with a possible distinct adrenal nodule phenotype (17), was missing in 27% of our cohort; while ever-smokers in our series showed larger baseline tumors, no association with hormonal findings or progression was observed. However, a key strength of our study is that it focuses exclusively on patients with discrete BAIs without radiological or clinical features suggestive of PBMAH at diagnosis, thereby providing more specific insights into the natural history of this distinct subgroup of patients.

Nevertheless, our findings suggest that patients with bilateral adrenal nodules may represent a distinct subgroup warranting individualized risk stratification and clinical reassessment. Prospective multicenter studies are needed to determine whether the observed biochemical and radiological progression translates into clinically meaningful outcomes (i.e., progression of comorbidities) and to define evidence-based follow-up strategies. Identifying patients at diagnosis who are more likely to progress over time may ultimately enable personalized follow-up strategies.

## Data Availability

The raw data supporting the conclusions of this article will be made available by the authors, without undue reservation.
